# The genome sequence of the nematode *Ostertagia ostertagi *(Stiles, 1892) (Rhabditida, Trichostrongylidae)

**DOI:** 10.12688/wellcomeopenres.26036.1

**Published:** 2026-03-19

**Authors:** Daniel R. G. Price, Yvonne Bartley, Margaret Oliver, Philip Steele, Marc Faber, Dave J. Bartley, Alison A. Morrison, Leigh Andrews, Alasdair J. Nisbet, Tom N. McNeilly, Neil A. Mabbott, Liam J. Morrison, Edwin Claerebout, Peter Geldhof, Lewis Stevens, Witold Morek, Dominic Absolon, Wellcome Sanger Institute Scientific Operations: Sequencing Operations, Mark Blaxter

**Affiliations:** 1 Department of Vaccines and Diagnostics, Moredun Research Institute, Edinburgh, Scotland, UK; 2 Department of Disease Control, Moredun Research Institute, Edinburgh, Scotland, UK; 3 Roslin Institute, Royal (Dick) School of Veterinary Studies, University of Edinburgh, Edinburgh, Scotland, UK; 4 Laboratory of Parasitology, Faculty of Veterinary Medicine, Ghent University, Merelbeke, Belgium; 5 Tree of Life Programme, Wellcome Sanger Institute, Hinxton, England, UK

**Keywords:** Ostertagia ostertagi, parasitic nematode, genome sequence, chromosomal, Rhabditida

## Abstract

We present a genome assembly from an individual nematode *Ostertagia ostertagi* (Rhabditida, Trichostrongylidae). The genome sequence has a total length of 407.17 megabases. Most of the assembly sequence (99.3%) was assigned to 6 chromosomal pseudomolecules, including the X sex chromosome. The mitochondrial genome has also been assembled, with a length of 14.49 kilobases.

## Species taxonomy

Eukaryota; Opisthokonta; Metazoa; Eumetazoa; Bilateria; Protostomia; Ecdysozoa; Nematoda; Chromadorea; Rhabditida; Rhabditina; Rhabditomorpha; Strongyloidea; Trichostrongylidae; *Ostertagia*; *Ostertagia ostertagi* Stiles, 1892 (NCBI:txid6317).

## Background

*Ostertagia ostertagi* (Rhabditina, Trichostrongylidae) is a parasitic nematode that primarily infects cattle, causing substantial economic losses to the dairy and beef industries ( Charlier *et al.*, 2020). It is commonly referred to as the “Brown stomach worm”. Adult parasites are small, slender and reddish brown in colour. Adult male and female worms measure 6–8 mm and 8–11 mm in length, respectively.

*O. ostertagi* has a direct lifecycle, with infective larvae (L3) ingested from pasture by grazing cattle. Third- and fourth-stage larvae reside in the gastric glands of the abomasum (the gastric stomach of cattle) with adult male and female parasites emerging from the glands and mating in the abomasal lumen. Infection in cattle is associated with the loss of acid-secreting parietal cells, an increase in gastric pH ( Murray *et al.*, 1970) and hypergastrinemia ( Fox *et al.*, 1987). Clinical signs of infection in cattle include parasitic gastroenteritis (PGE), reduced feed intake and gastrointestinal dysfunction ( Fox, 1993).

Here, we present a chromosome-level genome sequence of *Ostertagia ostertagi*, generated from a single male specimen of the *Ostertagia ostertagi* isolate MOo2, originally collected in Dudzele, West Flanders, Belgium.

## Methods

### Animal ethics

All procedures to gather samples from animals were approved by the Moredun Animal Welfare and Ethical Review Body (AWERB) and were conducted under the legislation of a UK Home Office License (reference P23F688B4) in accordance with the Animals (Scientific Procedures) Act of 1986.

### Sample acquisition

The *O. ostertagi* isolate, designated MOo2, was initially collected from cattle in Dudzele, West Flanders, Belgium, in 1996. The isolate was transferred to the Moredun Research Institute in 2012 and has since been maintained through annual passage in cattle kept under helminth-free conditions.

Helminth-free Holstein-Friesian male calves (4–10 months old) were challenged orally with 50,000 third-stage *O. ostertagi* larvae (L3). Individual male worms were collected from the abomasum of experimentally infected cattle at 21 days post infection (dpi). In brief, at post-mortem, the abomasa of the infected animals were opened along the greater curvature and washed in physiological saline (0.85% NaCl (w/v)), which was then poured over a 212 µm sieve. The sieve and contents were suspended in physiological saline at 37°C for four hours and the *O. ostertagi* parasites that migrated through the sieve were collected. Adult male worms were subsequently identified morphologically under a dissecting microscope ( MAFF, 1986) and individually picked into Eppendorf tubes before being frozen at -70°C.

To collect pools for Hi-C sequencing, L3 parasites were generated by coproculture from artificially infected *O. ostertagi* donor animals and isolated by passing through a Baermann apparatus according to Thienpont *et al.* (1986).

### Nucleic acid extraction

The PiMmS protocol ( Laumer, 2022) was used to extract and amplify the DNA from a male individual *O. ostertagi* (nxOstOste4). The extracted DNA was sheared to an average size of 10 kb using a Megaruptor 3 (Diagenode) using a shearing speed of 36. The sheared DNA was then amplified using 16 cycles of long-range PCR amplification with the Terra PCR Direct Polymerase (Takara Bio). The concentration of the sheared and purified DNA was assessed using a Qubit Fluorometer using the Qubit dsDNA High Sensitivity Assay kit (Thermo Fisher Scientific). Fragment size distribution was evaluated by running the sample on the TapeStation using Genomic DNA ScreenTape and reagents (Agilent Technologies).

### PacBio HiFi library preparation and sequencing

Libraries were prepared using the SMRTbell Prep Kit 3.0 (Pacific Biosciences) according to the manufacturer’s instructions. The kit includes reagents for end repair/A-tailing, adapter ligation, post-ligation SMRTbell bead clean-up, and nuclease treatment. Size selection and clean-up were performed using diluted AMPure PB beads (Pacific Biosciences). DNA concentration was quantified using a Qubit Fluorometer v4.0 (Thermo Fisher Scientific) and the Qubit 1X dsDNA HS assay kit. Final library fragment size was assessed with the Agilent Femto Pulse Automated Pulsed Field CE Instrument (Agilent Technologies) using the gDNA 55 kb BAC analysis kit.

The sample was sequenced using the Sequel IIe system (Pacific Biosciences, California, USA). The concentration of the library loaded onto the Sequel IIe was in the range 40–135 pM. The SMRT link software, a PacBio web-based end-to-end workflow manager, was used to set-up and monitor the run, and to perform primary and secondary analysis of the data upon completion.

### Hi-C sample preparation and crosslinking

Hi-C data were generated from a pool of L3 stage parasites (nxOstOste7) using the Arima-HiC v2 kit (Arima Genomics). Tissue was finely ground using the Covaris cryoPREP Dry Pulverizer (Covaris), and then subjected to nuclei isolation. Nuclei were isolated using a modified protocol based on the Qiagen QProteome Cell Compartment Kit (Qiagen), in which only the Lysis and CE2 buffers were used, with QIAshredder spin columns. After isolation, nuclei were fixed using formaldehyde to a final concentration of 2% to crosslink the DNA. The crosslinked DNA was then digested and biotinylated according to the manufacturer’s instructions. A clean-up step was performed with SPRIselect beads before library preparation. DNA concentration was quantified using the Qubit Fluorometer v4.0 (Thermo Fisher Scientific) and the Qubit HS Assay Kit, following the manufacturer’s instructions.

### Hi-C library preparation and sequencing

Biotinylated DNA constructs were fragmented using a Covaris E220 sonicator and size selected to 400–600 bp using SPRISelect beads. DNA was enriched with Arima-HiC v2 kit Enrichment beads. End repair, A-tailing, and adapter ligation were carried out with the NEBNext Ultra II DNA Library Prep Kit (New England Biolabs), following a modified protocol where library preparation occurs while DNA remains bound to the Enrichment beads. Library amplification was performed using KAPA HiFi HotStart mix and a custom Unique Dual Index (UDI) barcode set (Integrated DNA Technologies). Depending on sample concentration and biotinylation percentage determined at the crosslinking stage, libraries were amplified with 10–16 PCR cycles. Post-PCR clean-up was performed with SPRISelect beads. Libraries were quantified using the AccuClear Ultra High Sensitivity dsDNA Standards Assay Kit (Biotium) and a FLUOstar Omega plate reader (BMG Labtech). Prior to sequencing, libraries were normalised to 10 ng/µL. Normalised libraries were quantified again and equimolar and/or weighted 2.8 nM pools were created. Pool concentrations were checked using the Agilent 4200 TapeStation (Agilent) with High Sensitivity D500 reagents before sequencing. Sequencing was performed using paired-end 150 bp reads on the Illumina NovaSeq 6000.

### Genome assembly

TruSeq adapter sequences were removed from the genomic PacBio HiFi reads using lima, and PCR duplicates were removed using pbmarkdup. Any remaining adapter sequences were removed with HiFiAdapterFilt ( Sim *et al.*, 2022). The reads were assembled using hifiasm ( Cheng *et al.*, 2021) using the --primary option. The mitochondrial genome was identified and annotated using MitoHiFi 2.2 ( Uliano-Silva *et al.*, 2023). Residual haplotypic duplication from each assembly was removed using purge_dups ( Guan *et al.*, 2020). To scaffold the purged assembly into chromosomes, 10% of the Hi-C reads were randomly subsampled using SAMtools ( Danecek *et al.*, 2021), aligned to the purged primary assembly using bwa-mem ( Li, 2013), filtered to remove PCR duplicates using picard, and scaffolded using YaHS ( Zhou *et al.*, 2023).

### Assembly curation

The assembly was decontaminated using the Assembly Screen for Cobionts and Contaminants (ASCC) pipeline. TreeVal was used to generate the flat files and maps for use in curation. Manual curation was conducted primarily in PretextView and HiGlass ( Kerpedjiev *et al.*, 2018). Scaffolds were visually inspected and corrected as described by ( Howe *et al.*, 2021). Manual corrections included 114 breaks, 82 joins, and 62 haplotig removals. The X chromosome was identified by read coverage. The curation process is documented at https://gitlab.com/wtsi-grit/rapid-curation . Juicebox ( Durand *et al.*, 2016) was used to generate a Hi-C contact map of the final assembly.

### Assembly analysis and quality assessment

The Merqury.FK tool ( Rhie *et al.*, 2020) was used to evaluate *k*-mer completeness and assembly quality for the primary and alternate haplotypes using the *k*-mer database ( *k* = 31) computed prior to genome assembly. The analysis outputs included assembly QV scores and completeness statistics.

The genome was analysed using the BlobToolKit pipeline, a Nextflow implementation of the earlier Snakemake version ( Challis *et al.*, 2020). The pipeline aligns PacBio reads using minimap2 ( Li, 2018) and SAMtools ( Danecek *et al.*, 2021) to generate coverage tracks. It runs BUSCO ( Manni *et al.*, 2021) using lineages identified by querying the GoaT database ( Challis *et al.*, 2023). For the three domain level lineages, BUSCO genes are aligned to the UniProt Reference Proteomes database ( UniProt Consortium, 2023) using DIAMOND blastp ( Buchfink *et al.*, 2021). The genome is divided into chunks based on the density of BUSCO genes from the closest taxonomic lineage, and each chunk is aligned to the UniProt Reference Proteomes database with DIAMOND blastx. Sequences without hits are chunked using seqtk and aligned to the NT database with blastn ( Altschul *et al.*, 1990). The BlobToolKit suite consolidates all outputs into a blobdir for visualisation. The BlobToolKit pipeline was developed using nf-core tooling ( Ewels *et al.*, 2020) and MultiQC ( Ewels *et al.*, 2016), with package management via Conda and Bioconda ( Grüning *et al.*, 2018), and containerisation through Docker ( Merkel, 2014) and Singularity ( Kurtzer *et al.*, 2017).

The distribution of Nigon elements, which represent the seven ancestral linkage groups in rhabditid nematodes ( Gonzalez de la Rosa *et al.*, 2021), was visualised by providing the ‘full_table.tsv’ file from the BUSCO nematoda_odb10 output to the R script vis_ALG.R (available at https://github.com/lstevens17/vis_ALG_gn ), using a window size of 2 Mb.

## Genome sequence report

### Sequence data

**Figure 1.**
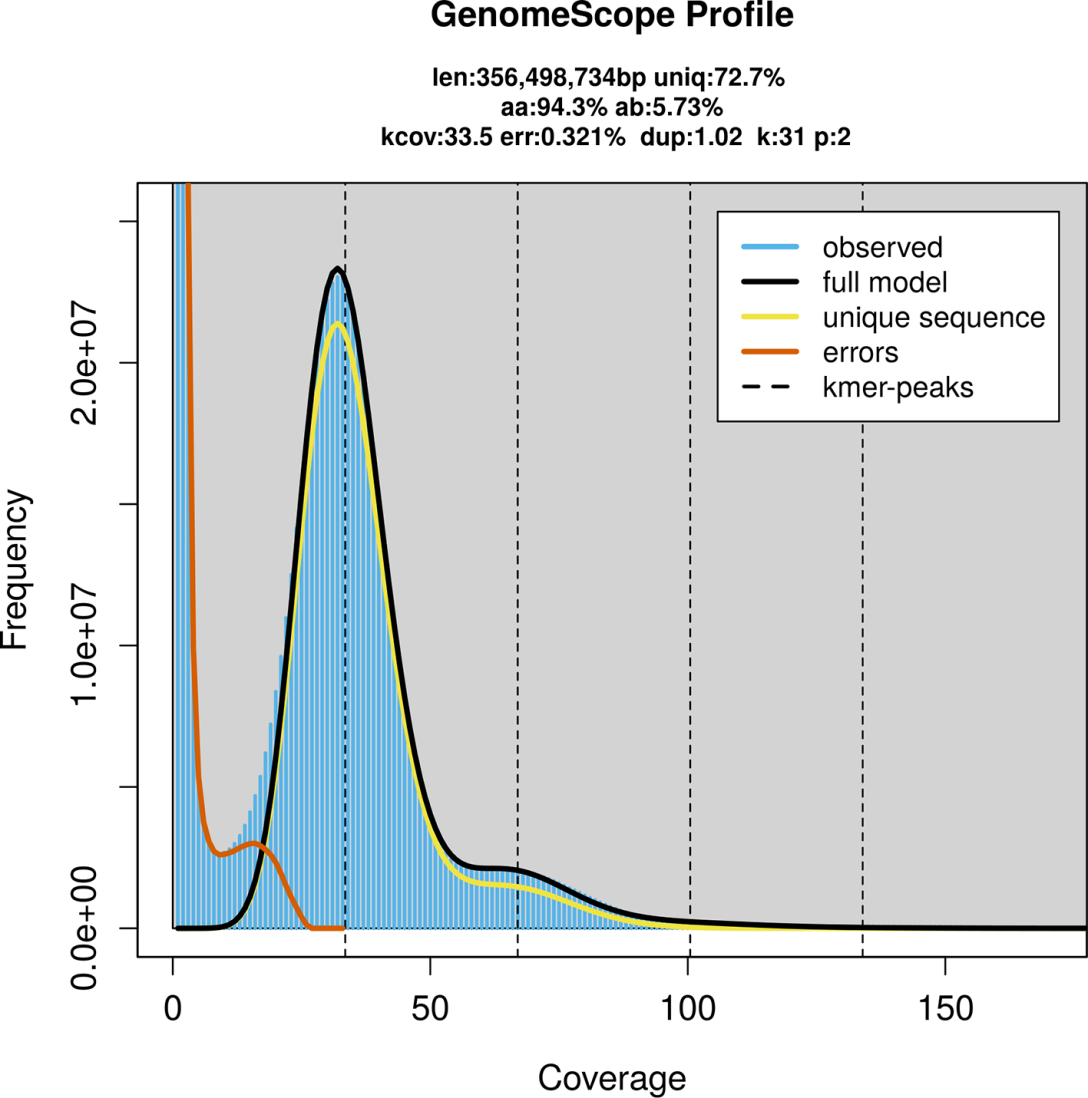
Frequency distribution of *k*-mer generated using GenomeScope2. The plot shows observed and modelled *k*-mer spectra, providing estimates of genome size, heterozygosity, and repeat content based on unassembled sequencing reads.

The genome of a male specimen of *O. ostertagi* was sequenced using Pacific Biosciences single-molecule HiFi long reads, generating 26.46 Gb (gigabases) from 2.74 million reads, which were used to assemble the genome. GenomeScope2.0 analysis estimated the haploid genome size at 356.50 Mb, with a heterozygosity of 5.73% and repeat content of 27.38% ( Figure 1). These estimates guided expectations for the assembly. Based on the estimated genome size, the sequencing data provided approximately 74× coverage. Hi-C sequencing produced 138.00 Gb from 913.95 million reads, which were used to scaffold the assembly. Table 1 summarises the specimen and sequencing details.

**Table 1.** Specimen and sequencing data.

Platform	PacBio HiFi	Hi-C
**ToLID**	nxOstOste4	nxOstOste7
**Specimen ID**	SAN20001109	SAN20002058
**BioSample (source individual)**	SAMEA110328943	SAMEA114558503
**BioSample (tissue)**	SAMEA110328955	SAMEA114558516
**Tissue**	whole organism	whole organism
**Instrument**	Sequel IIe	Illumina NovaSeq 6000
**Run accessions**	ERR12814183	ERR12814181
**Read count total**	2.74 million	913.95 million
**Base count total**	26.46 Gb	138.01 Gb

### Assembly statistics

The primary haplotype was assembled, and contigs corresponding to an alternate haplotype were also deposited in INSDC databases. The final assembly has a total length of 407.17 Mb in 117 scaffolds, with 767 gaps, and a scaffold N50 of 71.39 Mb ( Table 2).

**Table 2.** Genome assembly statistics.

Assembly name	nxOstOste4.1
Assembly accession	GCA_964213955.1
Alternate haplotype accession	GCA_964213895.1
Assembly level	chromosome
Span (Mb)	407.17
Number of chromosomes	6
Number of contigs	884
Contig N50	0.83 Mb
Number of scaffolds	117
Scaffold N50	71.39 Mb
Organelles	Mitochondrion: 14.49 kb

**Figure 2.**
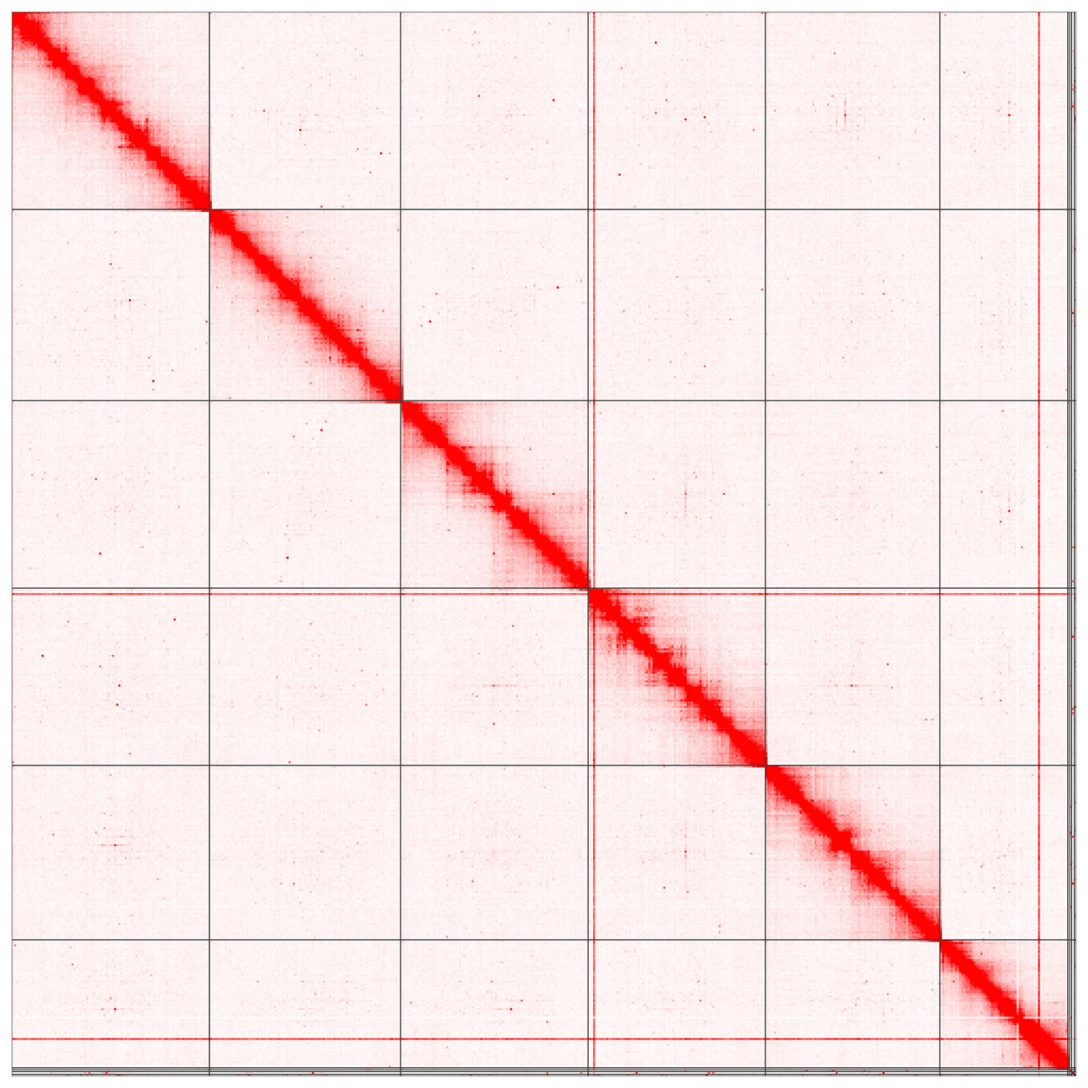
Hi-C contact map of the *Ostertagia ostertagi* genome assembly. Assembled chromosomes are shown in order of size. The plot was generated using Juicebox.

Most of the assembly sequence (99.3%) was assigned to 6 chromosomal-level scaffolds, representing 5 autosomes and the X sex chromosome. These chromosome-level scaffolds, confirmed by Hi-C data, are named according to size ( Figure 2; Table 3).

**Table 3.** Chromosomal pseudomolecules in the primary genome assembly of *Ostertagia ostertagi* nxOstOste4.

INSDC accession	Molecule	Length (Mb)	GC%
OZ172662.1	1	76.05	44.5
OZ172663.1	2	73.35	44.5
OZ172664.1	3	71.39	44.5
OZ172665.1	4	67.72	45
OZ172666.1	5	66.84	45
OZ172667.1	X	48.92	44.5

The mitochondrial genome was also assembled. This sequence is included as a contig in the multifasta file of the genome submission and as a standalone record.

**Figure 3.**
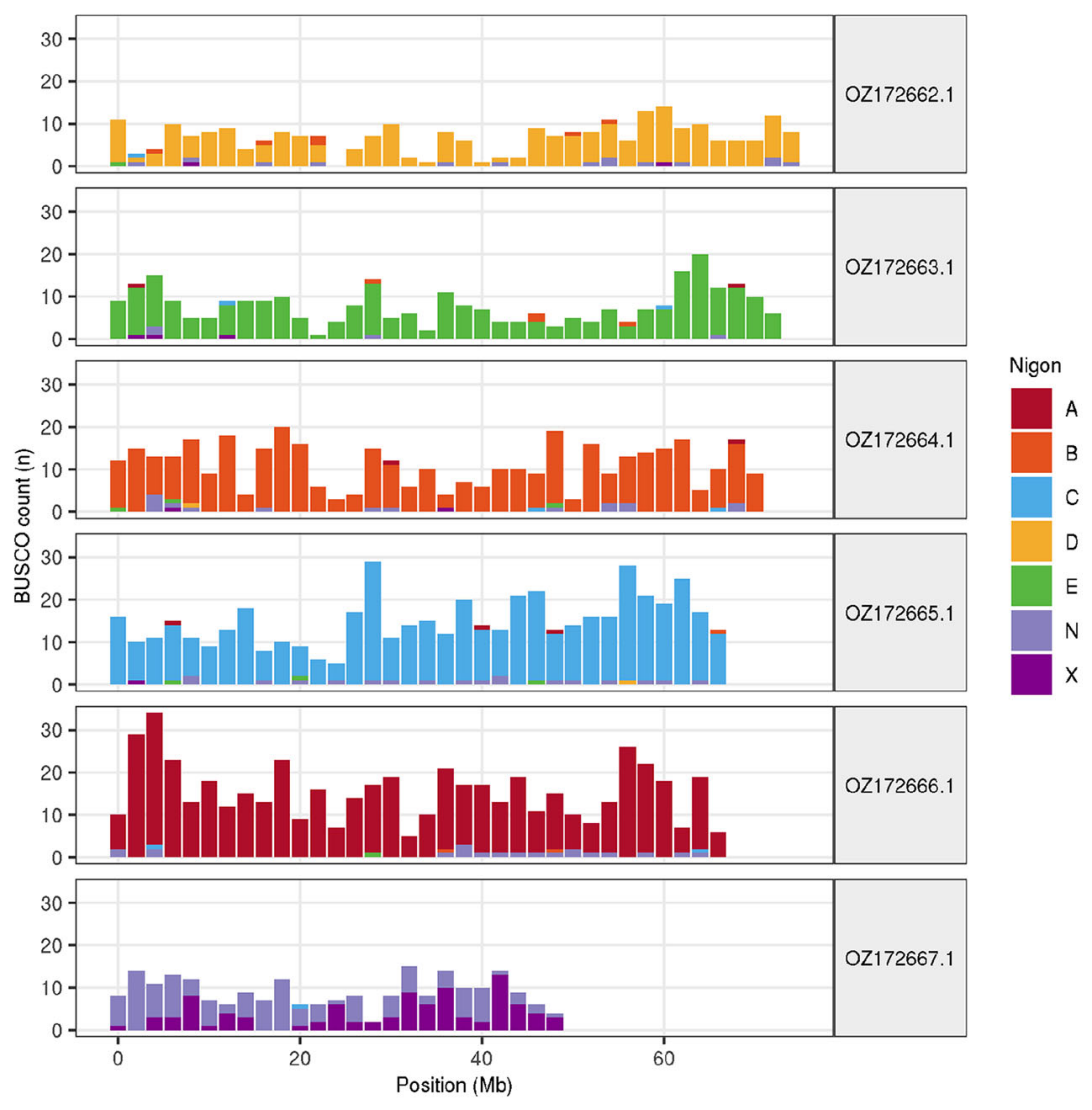
Nigon element painting of chromosomes in the nxOstOste4.1 assembly of *Ostertagia ostertagi.* Counts of Benchmarking using Single Copy Orthologues (BUSCO) loci in 2 Mb windows are coloured by their allocation to the seven Nigon elements (A-E, N, X).

Chromosome painting with Nigon elements illustrates the distribution of orthologues along chromosomes and highlights patterns of chromosomal evolution relative to ancestral linkage groups ( Figure 3).

### Assembly quality metrics

**Figure 4.**
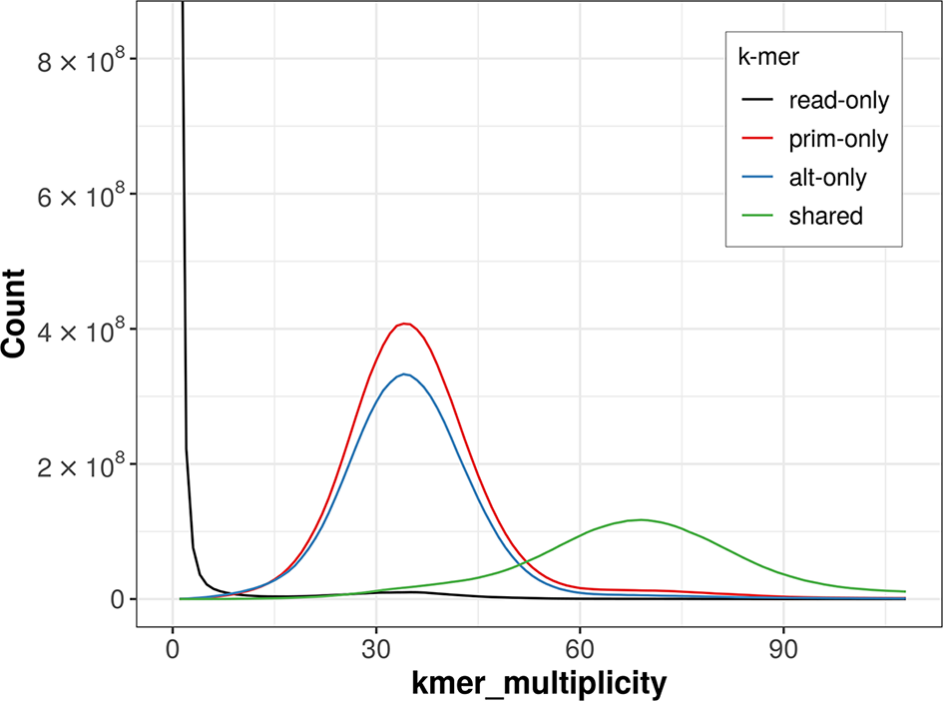
Evaluation of *k*-mer completeness using MerquryFK. This plot illustrates the recovery of *k*-mers from the original read data in the final assemblies. The horizontal axis represents *k*-mer multiplicity, and the vertical axis shows the number of *k*-mers. The black curve represents *k*-mers that appear in the reads but are not assembled. The green curve corresponds to *k*-mers shared by both haplotypes, and the red and blue curves show *k*-mers found only in one of the haplotypes.

The combined primary and alternate assemblies achieve an estimated QV of 56.9. The *k*-mer completeness is 59.87% for the primary assembly, 51.65% for the alternate haplotype, and 98.26% for the combined assemblies ( Figure 4).

**Figure 5.**
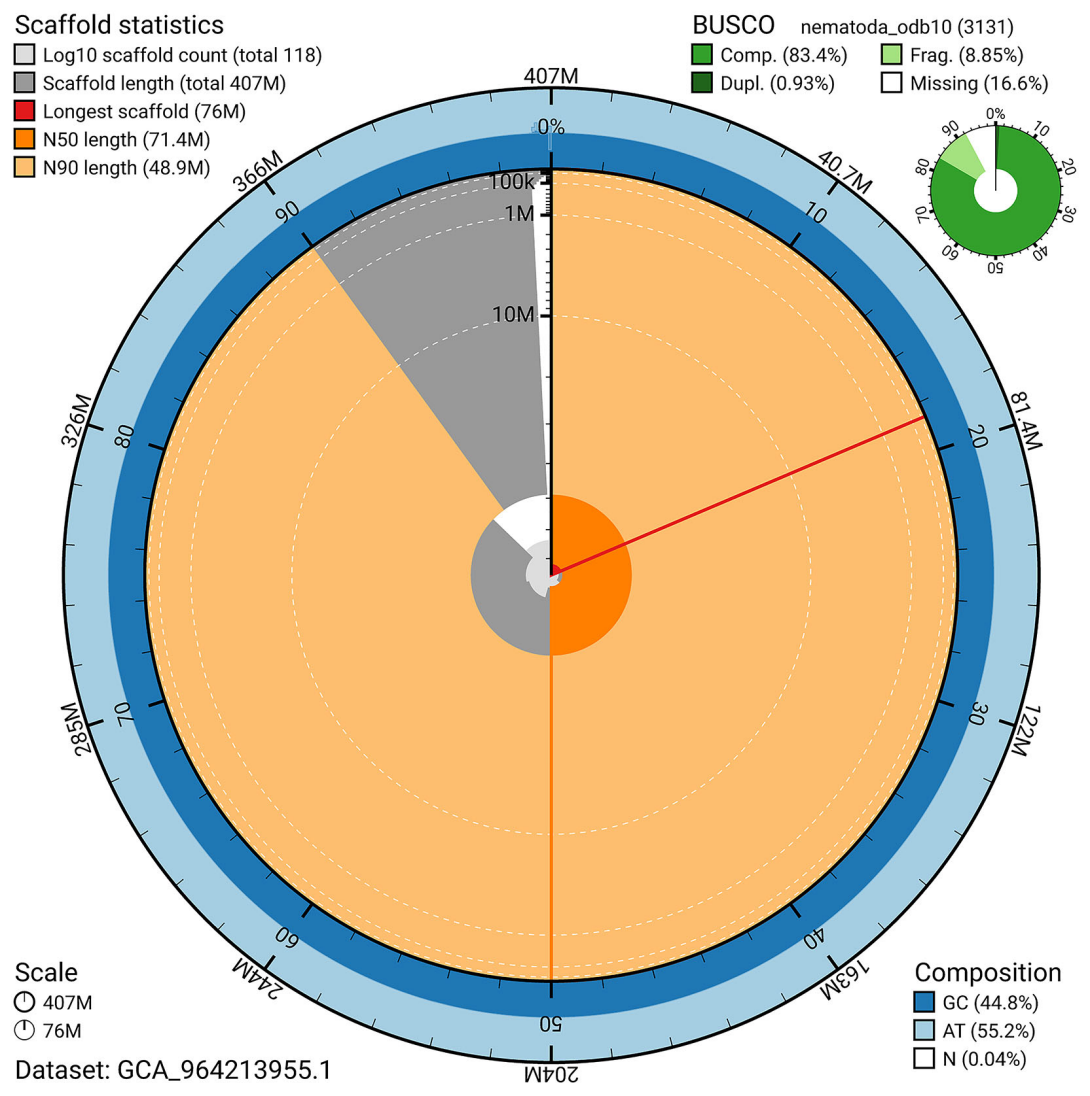
Assembly metrics for nxOstOste4.1. The BlobToolKit snail plot provides an overview of assembly metrics and BUSCO gene completeness. The circumference represents the length of the whole genome sequence, and the main plot is divided into 1,000 bins around the circumference. The outermost blue tracks display the distribution of GC, AT, and N percentages across the bins. Scaffolds are arranged clockwise from longest to shortest and are depicted in dark grey. The longest scaffold is indicated by the red arc, and the deeper orange and pale orange arcs represent the N50 and N90 lengths. A light grey spiral at the centre shows the cumulative scaffold count on a logarithmic scale. A summary of complete, fragmented, duplicated, and missing BUSCO genes in the nematoda_odb10 set is presented at the top right.

**Figure 6.**
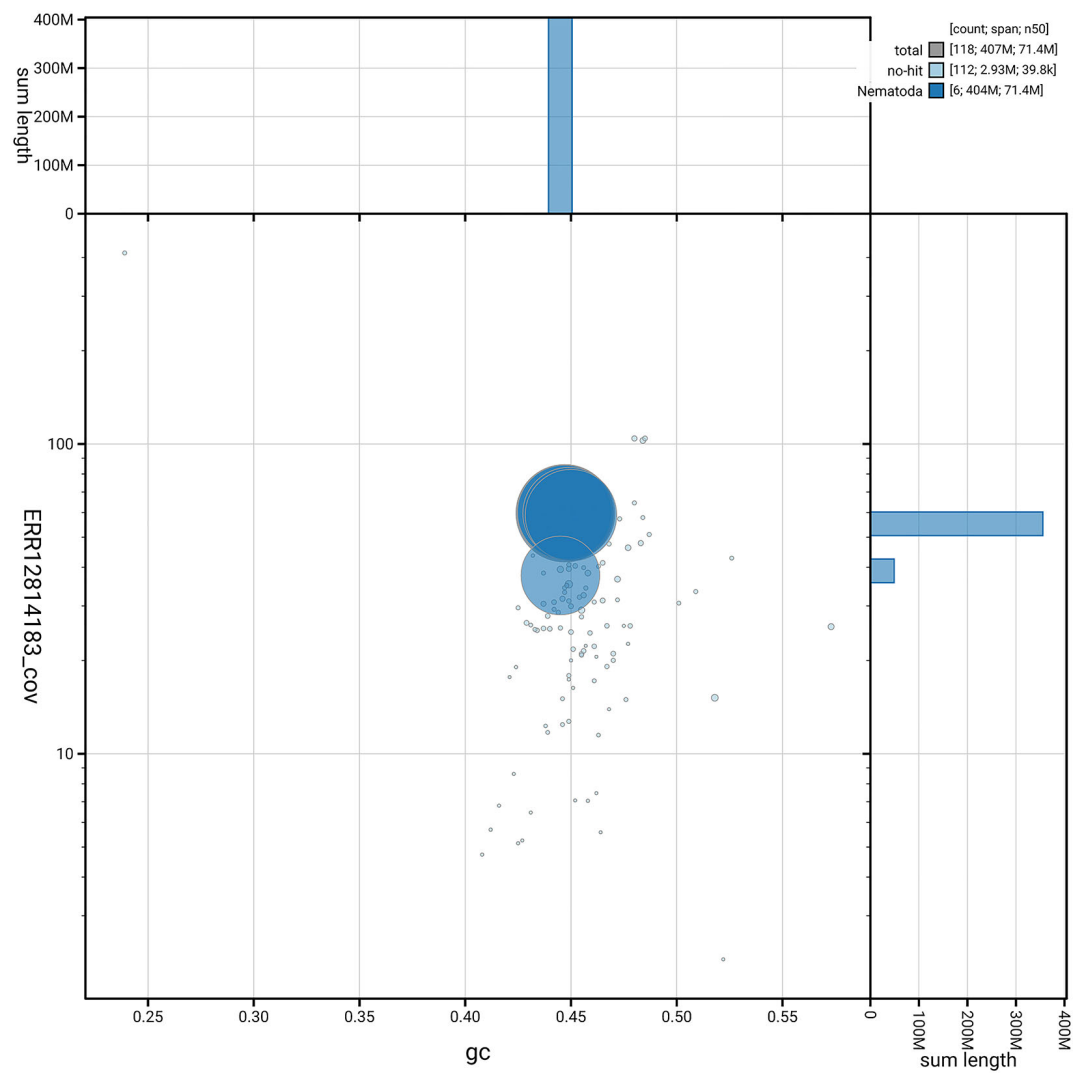
BlobToolKit GC-coverage plot for nxOstOste4.1. Blob plot showing sequence coverage (vertical axis) and GC content (horizontal axis). The circles represent scaffolds, with the size proportional to scaffold length and the colour representing phylum membership. The histograms along the axes display the total length of sequences distributed across different levels of coverage and GC content.

BUSCO v.5.7.1 analysis using the nematoda_odb10 reference set (n = 3131; Kriventseva *et al.*, 2019) identified 83.4% of the expected gene set (single = 82.5%, duplicated = 0.9%). The snail plot in Figure 5 summarises the scaffold length distribution and other assembly statistics for the primary assembly. The blob plot in Figure 6 shows the distribution of scaffolds by GC proportion and coverage.

Table 4 lists the assembly metric benchmarks adapted from Rhie *et al.* (2021) the Earth BioGenome Project Report on Assembly Standards September 2024. The EBP metric calculated for the primary assembly is 5.C.Q56, meeting the recommended reference standard for species with limited input material per individual.

**Table 4.** Earth Biogenome Project summary metrics for the *Ostertagia ostertagi* assembly.

Measure	Value	Benchmark
EBP summary (primary)	5.C.Q56	5.C.Q40
Contig N50 length	0.83 Mb	≥ 1 Mb
Scaffold N50 length	71.39 Mb	= chromosome N50
Consensus quality (QV)	Primary: 56.8; alternate: 57.0; combined: 56.9	≥ 40
*k*-mer completeness	Primary: 59.87%; alternate: 51.65%; combined: 98.26%	≥ 95%
BUSCO	C:83.4%[S:82.5%,D:0.9%], F:8.8%,M:7.8%,n:3131	S > 90%; D < 5%
Percentage of assembly assigned to chromosomes	99.3	≥ 90%

### Wellcome Sanger Institute – Legal and Governance

The materials that have contributed to this genome note have been supplied by a Tree of Life collaborator. The Wellcome Sanger Institute employs a process whereby due diligence is carried out proportionate to the nature of the materials themselves, and the circumstances under which they have been/are to be collected and provided for use. The purpose of this is to address and mitigate any potential legal and/or ethical implications of receipt and use of the materials as part of the research project, and to ensure that in doing so, we align with best practice wherever possible. The overarching areas of consideration are:

• Ethical review of provenance and sourcing of the material
• Legality of collection, transfer and use (national and international).

Each transfer of samples is undertaken according to a Research Collaboration Agreement or Material Transfer Agreement entered into by the Tree of Life collaborator, Genome Research Limited (operating as the Wellcome Sanger Institute), and in some circumstances, other Tree of Life collaborators.

## Data Availability

European Nucleotide Archive: *Ostertagia ostertagi.* Accession number PRJEB87158. The genome sequence is released openly for reuse. The *Ostertagia ostertagi* genome sequencing initiative is part of the Sanger Institute Tree of Life Programme (PRJEB43745) and 959 Nematode Genomes project (PRJEB81973). All raw sequence data and the assembly have been deposited in INSDC databases. Raw data and assembly accession identifiers are reported in Table 1 and Table 2. Pipelines used for genome assembly at the WSI Tree of Life are available at https://pipelines.tol.sanger.ac.uk/pipelines. Table 5 lists software versions used in this study.

## References

[ref1] Altschul SF Gish W Miller W : Basic Local Alignment Search Tool. *J Mol Biol.* 1990;215(3):403–410. 10.1016/S0022-2836(05)80360-2 2231712

[ref2] Buchfink B Reuter K Drost HG : Sensitive protein alignments at Tree-of-Life scale using DIAMOND. *Nat Methods.* 2021;18(4):366–368. 10.1038/s41592-021-01101-x 33828273 PMC8026399

[ref3] Challis R Kumar S Sotero-Caio C : Genomes on a Tree (GoaT): a versatile, scalable search engine for genomic and sequencing project metadata across the eukaryotic Tree of Life [version 1; peer review: 2 approved]. *Wellcome Open Res.* 2023;8:24. 10.12688/wellcomeopenres.18658.1 36864925 PMC9971660

[ref4] Challis R Richards E Rajan J : BlobToolKit - interactive quality assessment of genome assemblies. *G3 (Bethesda).* 2020;10(4):1361–1374. 10.1534/g3.119.400908 32071071 PMC7144090

[ref5] Charlier J Rinaldi L Musella V : Initial assessment of the economic burden of major parasitic helminth infections to the ruminant livestock industry in Europe. *Prev Vet Med.* 2020;182: 105103. 10.1016/j.prevetmed.2020.105103 32750638

[ref6] Cheng H Concepcion GT Feng X : Haplotype-resolved *de novo* assembly using phased assembly graphs with hifiasm. *Nat Methods.* 2021;18(2):170–175. 10.1038/s41592-020-01056-5 33526886 PMC7961889

[ref7] Danecek P Bonfield JK Liddle J : Twelve years of SAMtools and BCFtools. *GigaScience.* 2021;10(2): giab008. 10.1093/gigascience/giab008 33590861 PMC7931819

[ref9] Durand NC Robinson JT Shamim MS : Juicebox provides a visualization system for Hi-C contact maps with unlimited zoom. *Cell Syst.* 2016;3(1):99–101. 10.1016/j.cels.2015.07.012 27467250 PMC5596920

[ref10] Ewels P Magnusson M Lundin S : MultiQC: summarize analysis results for multiple tools and samples in a single report. *Bioinformatics.* 2016;32(19):3047–3048. 10.1093/bioinformatics/btw354 27312411 PMC5039924

[ref11] Ewels PA Peltzer A Fillinger S : The nf-core framework for community-curated bioinformatics pipelines. *Nat Biotechnol.* 2020;38(3):276–278. 10.1038/s41587-020-0439-x 32055031

[ref12] Fox MT : Pathophysiology of infection with *Ostertagia ostertagi* in cattle. *Vet Parasitol.* 1993;46(1–4):143–158. 10.1016/0304-4017(93)90055-r 8484207

[ref13] Fox MT Gerrelli D Pitt SR : Endocrine effects of a single infection with *Ostertagia ostertagi* in the calf. *Int J Parasitol.* 1987;17(6):1181–1185. 10.1016/0020-7519(87)90170-6 3308726

[ref8] Gonzalez de la Rosa PM Thomson M Trivedi U : A telomere-to-telomere assembly of *Oscheius tipulae* and the evolution of rhabditid nematode chromosomes. *G3 (Bethesda).* 2021;11(1): jkaa020. 10.1093/g3journal/jkaa020 33561231 PMC8022731

[ref14] Grüning B Dale R Sjödin A : Bioconda: sustainable and comprehensive software distribution for the life sciences. *Nat Methods.* 2018;15(7):475–476. 10.1038/s41592-018-0046-7 29967506 PMC11070151

[ref15] Guan D McCarthy SA Wood J : Identifying and removing haplotypic duplication in primary genome assemblies. *Bioinformatics.* 2020;36(9):2896–2898. 10.1093/bioinformatics/btaa025 31971576 PMC7203741

[ref16] Howe K Chow W Collins J : Significantly improving the quality of genome assemblies through curation. *GigaScience.* 2021;10(1): giaa153. 10.1093/gigascience/giaa153 33420778 PMC7794651

[ref17] Kerpedjiev P Abdennur N Lekschas F : HiGlass: web-based visual exploration and analysis of genome interaction maps. *Genome Biol.* 2018;19(1): 125. 10.1186/s13059-018-1486-1 30143029 PMC6109259

[ref18] Kriventseva EV Kuznetsov D Tegenfeldt F : OrthoDB v10: sampling the diversity of animal, plant, fungal, protist, bacterial and viral genomes for evolutionary and functional annotations of orthologs. *Nucleic Acids Res.* 2019;47(D1):D807–D811. 10.1093/nar/gky1053 30395283 PMC6323947

[ref19] Kurtzer GM Sochat V Bauer MW : Singularity: scientific containers for mobility of compute. *PLoS One.* 2017;12(5): e0177459. 10.1371/journal.pone.0177459 28494014 PMC5426675

[ref20] Laumer C : Picogram input multimodal sequencing (PiMmS) v1. 2022. 10.17504/protocols.io.rm7vzywy5lx1/v1

[ref21] Li H : Aligning sequence reads, clone sequences and assembly contigs with BWA-MEM.arXiv [q-bio.GN].2013. 10.48550/arXiv.1303.3997

[ref22] Li H : Minimap2: pairwise alignment for nucleotide sequences. *Bioinformatics.* 2018;34(18):3094–3100. 10.1093/bioinformatics/bty191 29750242 PMC6137996

[ref23] Manni M Berkeley MR Seppey M : BUSCO: assessing genomic data quality and beyond. *Curr Protoc.* 2021;1(12):e323. 10.1002/cpz1.323 34936221

[ref24] Merkel D : Docker: lightweight Linux containers for consistent development and deployment. *Linux J.* 2014;2014(239):2. Reference Source

[ref25] Ministry of Agriculture, Fisheries and Food (MAFF): Manual of veterinary parasitological laboratory techniques, Reference Book 418.Her Majesty’s Stationery Office 3rd edition,1986. Reference Source

[ref26] Murray M Jennings FW Armour J : Bovine ostertagiasis: structure, function and mode of differentiation of the bovine gastric mucosa and kinetics of the worm loss. *Res Vet Sci.* 1970;11(5):417–427. 10.1016/S0034-5288(18)34269-3 5276644

[ref27] Rhie A Walenz BP Koren S : Merqury: reference-free quality, completeness, and phasing assessment for genome assemblies. *Genome Biol.* 2020;21(1): 245. 10.1186/s13059-020-02134-9 32928274 PMC7488777

[ref33] Rhie A McCarthy SA Fedrigo O : Towards complete and error-free genome assemblies of all vertebrate species. *Nature.* 2021;592(7856):737–746. 10.1038/s41586-021-03451-0 33911273 PMC8081667

[ref28] Sim SB Corpuz RL Simmonds TJ : HiFiAdapterFilt, a memory efficient read processing pipeline, prevents occurrence of adapter sequence in PacBio HiFi reads and their negative impacts on genome assembly. *BMC Genomics.* 2022;23(1): 157. 10.1186/s12864-022-08375-1 35193521 PMC8864876

[ref29] Thienpont D Rochette F Vanparijs O : Diagnosing helminthiasis by coprological examination. 1986. Reference Source

[ref30] Uliano-Silva M Ferreira JGRN Krasheninnikova K : MitoHiFi: a python pipeline for mitochondrial genome assembly from PacBio high fidelity reads. *BMC Bioinformatics.* 2023;24(1): 288. 10.1186/s12859-023-05385-y 37464285 PMC10354987

[ref31] UniProt Consortium: UniProt: the Universal Protein Knowledgebase in 2023. *Nucleic Acids Res.* 2023;51(D1):D523–D531. 10.1093/nar/gkac1052 36408920 PMC9825514

[ref32] Zhou C McCarthy SA Durbin R : YaHS: Yet another Hi-C Scaffolding tool. *Bioinformatics.* 2023;39(1): btac808. 10.1093/bioinformatics/btac808 36525368 PMC9848053

