# Supplementary material for: The genome sequence of the nematode Ostertagia ostertagi (Stiles, 1892) (Rhabditida, Trichostrongylidae)
Source: Wellcome Open Res. 2026 Mar 19;11:187. [Version 1] doi: 10.12688/wellcomeopenres.26036.1 (PMC13139848; doi:10.12688/wellcomeopenres.26036.1)
Supplement: Peer review history

# Reviewer report: Reviewer response for version 1

**DOI:** 10.21956/wellcomeopenres.28670.r151406

**Published:** 2026-05-06

**Authors:** Neil David Young

**Affiliations:** 1 The University of Melbourne, Victoria, Australia

This reference genome is a welcome inclusion to the available nematode genomes that cause parasitic disease in livestock. The publication of this genome will allow important comparative genomic analyses among clade V nematodes. It is a shame that a gene set is not available upon indexing , but i assume this will be available in the near future. 

 Data and methods are current "best practice" and the metrics reported certainly indicate a complete reference genome for this species.

Are sufficient details of methods and materials provided to allow replication by others?

Yes

Is the rationale for creating the dataset(s) clearly described?

Yes

Are the datasets clearly presented in a useable and accessible format?

Yes

Are the protocols appropriate and is the work technically sound?

Yes

Reviewer Expertise:

Parasitology, Genomics

I confirm that I have read this submission and believe that I have an appropriate level of expertise to confirm that it is of an acceptable scientific standard.

# Reviewer report: Reviewer response for version 1

**DOI:** 10.21956/wellcomeopenres.28670.r154569

**Published:** 2026-05-04

**Authors:** Erich M. Schwarz

**Affiliations:** 1 Cornell University, Ithaca, New York, USA

Price et al. provide a chromosomal-quality reference genome for Ostertagia ostertagi, a trichostrongylid nematode parasite of cattle with longstanding relevance to veterinary medicine and agriculture.

 The paper is generally satisfactory, with one minor question:

 What is the reference "MAFF, 1986" that the authors cite for identifying adult males morphologically under a dissecting microscope?  This citation is missing from the References section.

Are sufficient details of methods and materials provided to allow replication by others?

Yes

Is the rationale for creating the dataset(s) clearly described?

Yes

Are the datasets clearly presented in a useable and accessible format?

Yes

Are the protocols appropriate and is the work technically sound?

Yes

Reviewer Expertise:

Nematode genomics

I confirm that I have read this submission and believe that I have an appropriate level of expertise to confirm that it is of an acceptable scientific standard.

# Reviewer report: Reviewer response for version 1

**DOI:** 10.21956/wellcomeopenres.28670.r152020

**Published:** 2026-04-11

**Authors:** Jun Kim

**Affiliations:** 1 Chungnam National University, Daejeon, Daejeon, South Korea

This study presents an important contribution by reporting a chromosome-level genome assembly of the parasitic nematode Ostertagia ostertagi. To overcome the limitations imposed by the small body size of this nematode, the authors generated long-read sequencing data using a whole-genome amplification-based approach and successfully obtained a high-quality genome assembly. Although there are several points for which additional detail would be helpful, the manuscript is, overall, already very well prepared. I would also like to express my appreciation to the Tree of Life team at the Wellcome Sanger Institute for consistently providing high-quality genome assembly resources to the research community.

 The following points may merit further clarification.

 Q1. It appears that whole-genome amplification was used. It would therefore be helpful to add a row in Table 1 indicating the sample type, for example whether the material was derived from a single individual or an L3 pool. In addition, information on the reads themselves is currently lacking. Providing the read length and quality distributions would be highly valuable for future researchers working on similarly small-bodied organisms.

 Q2. Although Figure 6 includes coverage information, it would be even more informative to provide this at higher resolution. Specifically, in addition to scaffold-level coverage, it would be useful to show read depth across each scaffold using smaller windows, for example 100 kb bins. Given that PCR-based whole-genome amplification was performed, some degree of GC bias is likely to be present. Information on this point could serve as an important reference for future studies.

 Q3. Since a chromosome-level genome assembly has been generated, it would be worthwhile to examine whether telomeric regions are present at the ends of each chromosome. Considering that the sequencing data were obtained following amplification, it is possible that telomeric repeats were not fully captured (REF1). I am therefore first curious whether all 12 telomeric regions were successfully recovered at the contig level. In addition, because mis-scaffolding could plausibly have occurred during the Hi-C scaffolding process, it would be helpful to confirm whether any telomeric regions are located internally within chromosomes.

 Q4. Finally, it appears that RNA-seq and gene annotation were not performed. I would appreciate it if the authors could confirm whether this is correct.

Are sufficient details of methods and materials provided to allow replication by others?

Yes

Is the rationale for creating the dataset(s) clearly described?

Yes

Are the datasets clearly presented in a useable and accessible format?

Yes

Are the protocols appropriate and is the work technically sound?

Yes

Reviewer Expertise:

Genomics

I confirm that I have read this submission and believe that I have an appropriate level of expertise to confirm that it is of an acceptable scientific standard.

## References

[1] : Telomeric repeat evolution in the phylum Nematoda revealed by high-quality genome assemblies and subtelomere structures. *Genome Research* .2023;33(11) : 10.1101/gr.278124.123 1947-1957 10.1101/gr.278124.123 PMC1076044937918961
